# Editorial of Special Issue Ruthenium Complex: The Expanding Chemistry of the Ruthenium Complexes

**DOI:** 10.3390/molecules200917244

**Published:** 2015-09-18

**Authors:** Ileana Dragutan, Valerian Dragutan, Albert Demonceau

**Affiliations:** 1 Romanian Academy, Institute of Organic Chemistry “C.D. Nenitescu”, Bucharest 060023, Romania; 2 Department of Chemistry, University of Liège, Sart-Tilman (B.6a), Liège 4000, Belgium

**Keywords:** ruthenium complexes, anticancer agents, catalysis, nanomaterials

## Abstract

Recent trends in Ru complex chemistry are surveyed with emphasis on the development of anticancer drugs and applications in catalysis, polymers, materials science and nanotechnology.

## 1. Introduction

Over the last couple of years, the field of coordination and organometallic chemistry of ruthenium has grown and evolved at unprecedented rates. Recent publications largely highlight key advances on Ru-based complexes and on their chemically addressable applications in challenging areas like biology, medicine, catalysis, nanoscience, redox and photoactive materials, *etc.* [1,2,3,4,5]. This explosive expansion is mainly due to the unique ability of the ruthenium core to permit multiple oxidation states, hence versatile electron-transfer pathways.

Both Ru(II) and Ru(III) oxidation states accommodate six-coordinated octahedral configurations in which the additional axial ligands help fine-tune the steric and electronic properties of the complexes. Of consequence are also the relative weakness of certain metal-ligand bonds as well as the thermodynamic and kinetic stability of Ru(III)-complexes *vs.* Ru(II)-complexes since ligand exchange kinetics are immediately affected [6]. Proper variations of ancillary ligands, allowing modulation of redox properties and ligand interactions, have resulted in a large platform of Ru complexes endowed with achiral or chiral configurations [7,8,9,10,11,12,13,14]. While older research takes new directions, cutting-edge applications have emerged in the already mentioned fields, yet also in materials science, petrochemistry, and oleochemistry. Further valorization of Ru complexes in photochemistry and photophysics, for pharmaceuticals and agrochemicals will undoubtedly be forthcoming. Admittedly, this brief overview cannot cover the huge diversity of recently introduced ruthenium complexes and concentrates essentially on concepts and trends disclosed during 2013–2014 and early 2015. A critical appraisal of these aspects is presented showcasing Ru-driven advances in anticancer chemotherapy, medicinal applications, catalysis, water oxidation, photoactive complexes, functional and nanomaterials and complex design that have newly taken center stage.

## 2. Ruthenium-Based Anticancer Drugs. Medicinal Applications

Though started over 50 years ago, the development of metal anticancer drugs has traditionally focused on cytotoxic platinum compounds, several of which have reached clinical application [15]. The deeper understanding of cancer biology triggered the introduction of targeted chemotherapies, using other metal-based drugs, able to address specific cancer physiologies or disease states [16,17,18,19]. As most of the ruthenium complexes are less toxic than their platinum counterparts, this progress offers considerable added value for medical implementation. Although so far none of the ruthenium complexes are in clinical use as anticancer drugs, the remarkable success in clinical trials of NAMI-A, KP1019, and KP1339, combined with abundant reports on the enhanced *in vitro* and *in vivo* activity of other types of ruthenium complexes, prompted ruthenium chemotherapeutics to rapidly become a major area in anticancer drug advancement [20,21,22,23,24,25,26]. In an illustrative example NAMI-A, inactive against primary tumors but specifically targeting tumor metastases, was shown to display an improved antimetastatic activity when turned into macromolecular NAMI-A, a biocompatible amphiphilic block copolymer forming micelles that increase the cytotoxicity and cell uptake of the drug [22]. In the search for superior metallodrugs, ruthenium chemistry is taking momentum through the introduction of Ru-containing macromolecular complexes (dendrimers, dendronized polymers, protein conjugates, intelligent nanoparticles for advanced drug delivery, and polymer Ru-complex conjugates) that better differentiate between tumor cells and healthy cells [26].

Currently considered among the most promising alternatives to platinum drugs, active by interaction with DNA, ruthenium complexes also operate via different mechanisms [27,28,29,30,31,32,33]. Both Ru(II) and Ru(III) oxidation states are stable in physiological solutions, with the latter considered to be less reactive; therefore, Ru complexes are generally reported to act as redox-activatable prodrugs [34,35]. Recent research largely illustrates that the *in vitro* and *in vivo* properties of ruthenium compounds can be finely tuned by ligand variation. Intense investigations converge on ligand selection from the arene, phosphine, aromatic heterocycles, pyridine, pta, pybox, pyrazolone-based β-ketoamine, nitrosyl, Schiff-base, carboxamide, carbothioamide, thiourea, thiocarbamate, thiosemicarbazone, and hydrazone classes [36,37,38,39,40,41,42,43,44,45,46,47,48,49,50,51,52,53,54,55,56,57,58,59,60,61,62,63,64,65,66,67,68,69,70,71,72,73,74,75,76,77,78]. Innovations were applied to construct coordination-driven self-assembled arene-Ru metalla-rectangles endowed with stronger cytotoxicity against all cancer cell lines and even more effective than established anticancer drugs like doxorubicin and cisplatin [50]. That interest in the bioactivity profiles of ruthenium complexes has grown rapidly is amply demonstrated by design of new Ru compounds inhibiting enzymes, by the novel multinuclear systems acting as drug delivery vehicles or imaging and theranostics agents and signal transduction elements. A major contribution came as well from the advent of refined bioanalytical, biophysical and spectroscopic techniques used to elucidate the structure and the *in vivo* functioning of these complexes [79,80,81,82,83,84,85,86,87,88,89,90,91,92,93,94,95,96,97,98,99,100]. Some arene-functionalized dinuclear organometallic Ru(II) complexes are capable of crosslinking model peptide and oligonucleotide sequences while cytotoxicities are linked to their more rigid or more flexible conformations [81]. The mechanism of action of ruthenium-based antitumor drugs was studied, especially in regard to the capacity of ruthenium to mimic iron in its binding to biological molecules. Along these lines, new ruthenium complexes containing the enantiopure ligands 2,6-bis[4′(*R*)-phenyloxazolin-2′-yl-pyridine] ((*R*,*R*)-Ph-pybox), 2,6-bis[4′(*S*)-isopropyloxazolin-2′-yl-pyridine] ((*S*,*S*)-*i*Pr-pybox) or 2,6-bis[4′(*R*)-isopropyloxazolin-2′-yl-pyridine] ((*R*,*R*)-*i*Pr-pybox) and the water soluble 1,3,5-triaza-7-phosphaadamantane (PTA) or *N*-substituted PTA phosphanes, have been synthesized. Their interactions with plasmidic DNA and cytotoxic activities against human cervical cancer HeLa cell lines have been evaluated, evidencing the distinct ability of the enantiomeric ligands in affecting the cell cycle of HeLa tumor cells [101].

Incorporation of biologically-derived ligands that primarily aim at minimizing toxicity toward normal cells is a viable avenue for engineering ruthenium complexes since they provide ways to improve antiproliferative activity of metal-based drugs [102]. Such ligands display different coordination modes, facilitate compatibility of the complex with the biological environment, and promote a higher cellular uptake. The diversity of bio-relevant ligands installed as essential components of ruthenium complexes ranges from amino acids, peptides, proteins, carbohydrates, purine bases and oligonucleotides to steroids and other bioactive entities endowed with specific properties [103,104,105,106,107,108]. This methodology is confirmed by excellent studies on Ru complexes that open unique opportunities for rational design and production of potent ruthenium anticancer drugs tackling distinct transport pathways and mechanisms of action [108,109].

Photoactivation of ruthenium complexes for triggering and/or modulating their antitumor activity [110,111,112,113] is presently of great interest. The process enables transformation of unreactive Ru(II) complexes into light-driven cytotoxic species [114] that can subsequently interact with proteins and DNA at the cellular level. In some representative examples, it has been demonstrated that, using a clinical grade photodynamic therapy laser source, red-to-blue upconverting liposomes were capable of triggering photodissociation of ruthenium polypyridyl complexes from PEGylated liposomes [115]. Several ruthenium(II) polypyridyl complexes have been elaborated to act as two-photon absorption (TPA) agents in mitochondria-targeted photodynamic therapy, a promising new technique for resolving tumors selectively and subduing resistance to alternative anticancer therapies [116]. Cellular toxicity induced by photorelease of certain bioactive molecules has been proved as a valid alternative for Ru photodynamic therapeutic agents (PDT) with dual-action [117,118,119]. 

When photoactivated, surface-grafted ruthenium complexes on mesoporous silica nanoparticles undergo photoexpulsion and subsequent release as cytotoxic entities toward cancer cells [120]. Nowadays, numerous accounts on photoactivatable Ru(II) complexes that exhibit different types of biologically relevant activities have been published [121,122,123,124,125]. Of real consequence for therapeutic purposes, catalytic reactions induced in cells by certain metallodrugs pertaining to the class of redox modulators or photosensitizers may ensure low-dose therapy protocols [126]. An interesting catalytic procedure demonstrated that transfer hydrogenation promoted by Noyori-type ruthenium complexes can reduce coenzyme NAD^+^ to NADH in human ovarian cancer cells by using non-toxic concentrations of formate as a hydride donor source [127]. Such advanced catalytic processes enhance capabilities of ruthenium complexes, while also increasing selectivity towards cancer cells *versus* normal cells.

Ruthenium complexes additionally find important medicinal applications as NO carriers and donors for induction of vascular relaxation (some Ru(III) complexes can act as nitric oxide scavengers thus improving graft survival). Drugs based on specific Ru complexes are active against parasitic diseases (e.g., leishmanicidal and protozoanicidal agents), as antibacterial, antifungal and anticorrosion additives in coatings on Ti-alloys used in medical devices [128,129,130,131,132,133,134].

## 3. Ruthenium Complexes in Catalysis

Unquestionably, the second major area of overwhelming importance is catalysis promoted by Ru complexes, as also recognized by the Nobel Prizes awarded for the field. Productive catalytic applications of these complexes embrace a broad variety of chemical transformations, have known tremendous recent progress, while some are valorized on large scale, have true industrial perspectives or have already been industrialized [135]. Noteworthy, environmentally friendly homogeneous and immobilized ruthenium catalysts enabled indispensable synthetic methods inaccessible with other metal-based catalytic systems. With the aim of upgrading versatility of the ruthenium-based catalysis, new mononuclear, binuclear and polynuclear ruthenium complexes with arene, phosphine, phosphite, BINAP, alkylidene, vinylidene, allenylidene, indenylidene, *N*-heterocyclic carbene (NHC), cyclic alkyl amino carbene (CAAC), porphyrin, pincer, Schiff-base, polydentated and polycyclic ligands have continued to be prepared, minutely characterized and explored in chemoselective catalytic processes [136,137,138,139,140,141,142,143,144,145,146,147,148,149,150,151,152,153,154,155,156,157,158]. In an innovatory undertaking, graphene oxide was used to support ruthenium catalysts in order to activate self-healing in multifunctional materials that are able to simultaneously integrate the healing process with the advantageous properties of graphene-based materials [159]. Part of a large body of work concerns fundamental reactions studied anew with Ru complexes including hydrogenation and transfer hydrogenation; oxidation and hydroxylation; C–C, C–X and N–X bond formation; olefin metathesis and related C–C couplings; alkylation; arylation; isomerization; epimerization; condensation; cyclization; atom transfer radical reactions; oligomerization and polymerization [160,161,162,163,164,165,166,167,168,169,170,171,172,173,174,175,176,177,178,179,180,181,182,183,184,185,186,187,188,189,190,191,192,193]. Among non-metathetical reactions, the versatile and easy to handle transfer hydrogenation is often chosen as standard method for appraising and comparing the catalytic activity of ruthenium promoters [167,168,169,170,171,172,173].

In the chase for renewable energy sources currently in high demand, hydrogen resulting from electrochemical water splitting has been proposed as an energy carrier. Not long ago, an ingenuous method for hydrogen storage has been disclosed. The process, based on a CO_2_(HCO_3_^−^)/H_2_ and formate equilibrium within an amine-free reversible system is catalyzed by commercially available Ru pincer complexes and produces an advantageous mixture of H_2_ (CO free) and CO_2_ [194]. As a valuable addition to the multiple Ru-based catalyses, bond activations and functionalizations (e.g. H–H, C–H, N–H, O–H, B–H, and Si–H) have been explored [195,196,197,198].

Stereoselective oxidative reactions of a host of functional groups, more specifically asymmetric epoxidation of alkenes, dihydroxylation of olefins, oxidative dehydrogenation of alcohols and generation of dioxygen species, have also been addressed using diverse ruthenium catalysts [199]. For example, a recent communication clearly evidenced that Ru–aqua complex {[Ru^II^(trpy)(H_2_O)]_2_(μ-pyr-dc)}^+^can be a powerful epoxidation catalyst for a wide range of linear and cyclic alkenes [200].

A subject of renewed interest is presently the production of oxygen through water splitting by help of Ru catalysts as showcased in excellent reviews [201,202,203]. Mononuclear and binuclear ruthenium complexes have been explored in this reaction outlining the influence of monodentate and polydentate ligands on the course of water oxidation [204,205,206,207,208,209,210,211,212]. In the opposite process, water reduction, conducted with polydentated Ru catalysts, the solar H_2_ production was coupled to photoinitiated electron collection in polyazine chromophores [213].

One recent refined approach applies Ru(bpy)_3_Cl_2_ as photoredox catalyst and *p*-toluidine as redox mediator in the visible light photocatalytic thiol−ene “click” reaction for fast polymer post-functionalization and step-growth polymerization, under aerobic conditions [214].

Schiff-base (e.g. Salen) Ru-complexes enable versatile asymmetric catalysis for a range of chemical transformations, especially for carbene, nitrene, and oxene transfer reactions [11,146,215,216]. Complexes of this class reportedly serve as catalysts for oxidation/epoxidation, with chiral Ru-complexes such as Ru-porphyrin, desymmetric Ru-Schiff base and Ru-bisamide complexes being employed in asymmetric epoxidation. Importantly, (ON^+^)(Salen)ruthenium(II) complexes were found to catalyze epoxidation of conjugated olefins under light irradiation. In some instances, these olefins showed high enantioselectivity, greater than 80% *ee*, irrespective of their substitution pattern.

Latterly, vast research centers on the functionalization of diverse organic substrates as promoted by ruthenium catalysts. For instance, alkynylation of aldehydes, hydroacylation, hydroboration and hydrosilylation of terminal alkynes, conjugate addition of alkynes to α,β-unsaturated carbonyl compounds, enantioselective carbonyl allylation, decarbonylation of acetone and carbonate, *N*-formylation of amines and reduction of hydrazine to ammonia (occurring with high chemoselectivity) have been tackled [198,217,218,219,220,221,222,223,224]. Racemization of *sec*-alcohols catalyzed by homogeneous half-sandwich ruthenium complexes (Cp*Ru(CO)_2_Cl) has been newly examined [225,226,227]; under the influence of the ruthenium catalyst, only the hydroxyl-substituted stereocenter was interconverted occasionally, making epimerization analogous to racemization. Computational investigations on CO dissociation within cyclopentadienyl ruthenium complexes, relevant to the racemization of alcohols, have been carried out as well [228]. For the 16- and 18-electron *p*-cymene ruthenium complexes, the ligand dissociation effect in racemization and dynamic kinetic resolution of alcohols was appraised [229]. A cationic ruthenium complex for the efficient dynamic kinetic resolution of secondary alcohols has also been communicated [230].

With introduction of the new generations of highly active and chemoselective Ru metathesis catalysts endowed with high tolerance towards many functionalities and good stability in the presence of air, moisture and various solvents, including water-containing systems [231,232,233,234,235,236,237,238,239], the diversification of metathesis reactions experienced a strong impetus [240,241,242,243,244,245]. Among metathesis reactions, cross-metathesis (CM) [246,247,248,249] and ring-closing metathesis (RCM) [250,251,252,253,254] have become the most encountered strategies for the synthesis of linear and branched, or carbocyclic and heterocyclic compounds. Ru-catalyzed enyne metathesis (EYRCM) provided an elegant and productive way to obtain small, medium and large cyclic molecules [255]. Numerous tandem metathesis protocols (RCM-CM, RCM-RCM, and RCM-ROM-CM), as well as related processes (C–C coupling, cyclopropanation, cyclizations, and polymerizations) facilitated the synthesis of complex organic molecules, intermediates for fine chemicals, bioactive natural products, macrocyclic or polycyclic structures, functional polymers and supramolecular architectures [256,257,258,259,260,261,262,263,264]. Intricate scaffolds for pharmaceuticals and agrochemicals have been accessed by these advanced strategies [265,266,267]. In a short time total syntheses, involving key metathesis steps (mostly RCM or EYRCM) were competitively advanced by different groups, providing an elegant and easier access to a plethora of complex bioactive molecules that display antitumor, antiviral, anti-HIV, antimicrobial, antifungal activities, and also to alkaloids and other classes of naturally occurring compounds [260,261,268]. More particular cases like metathesis-based syntheses of a range of iminosugars, and bioactive iriomoteolide and kendomycin were given special attention [269,270,271,272,273]. Synthesis, involving enyne metathesis, of biologically and medicinally significant molecules like (+)-anatoxin-A, (+)-anthramycin, artemisinin, transtaganolide D, allo-colchicine, (−)-galanthamine, (−)-longithorone A, (+)-ochromycinone, (+)-rubiginone B2, and (−)-stemoamide have become attractive when starting from adequate substrates [274]. It should be emphasized that cross-metathesis allowed diversification of synthetic strategies by employing substrates that are tolerated by the new generations of Ru complexes [231,236,237,238,239,243,244,246,247,248,249]. A remarkably creative work encompasses the total synthesis of (−)-amphidinolide K and V applying an ingenious combination of Mo with Ru metathesis catalytic systems [275].

Ring-opening metathesis polymerization (ROMP) of monocyclic, bicyclic and polycyclic monomers with Ru complexes [276,277,278,279] unveiled polymerization methodologies capable to deliver unique materials not available by conventional polymerization techniques [280,281,282,283,284,285]. New types of telechelic and supramolecular polymers have been obtained by devising ruthenium catalysts suitable for their synthesis [286,287]. Ru complexes also intervene in acyclic diene metathesis polymerization (ADMET) [288,289] and polymerization of substituted acetylenes [290]. Not long ago, novel synthetic procedures based on ruthenium catalysis extended the range of biologically active polymers, biorenewable polymers and biodegradable copolymers [291,292,293].

Materials science took advantage of the contemporary capabilities of Ru complexes in inventive applications destined for industrial technologies. Thus, syntheses of nanoparticles and materials with nanostructured periodicity have been elaborated [294,295]. Polydicyclopentadiene (pDCPD) aerogels, obtained via ROMP, that are low-density nanostructured nanoporous solids with high surface-to-volume ratios, target applications including thermal and acoustic insulation, porous membranes, superhydrophobic surfaces and antireflection coatings [296]. Nanostructures based on gold-anchored ROMP polymers were introduced as valuable materials for microelectronics, catalysis and optophysics.

With the purpose of enhancing the photovoltaic performance of solar cells, different research groups have attempted to modify the structure of the coordinated chromophoric and ancillary ligands of ruthenium-based dye sensitizers [297,298,299,300]. For example, though initially ruthenium(II) complexes used in luminescent applications such as dye-sensitized solar cells incorporated only poly-pyridine, poly-azolium or other poly-*N*-heterocyclic moieties, of late NHC substituents have also been added to give rise to the high energy emissions required for OLED devices [301]. In this new context, it has been shown that the free rotation in polypyridine ligands of Ru(II) complexes induces a substantial effect on the performance of light-emitting electrochemical cells [302]. Advanced research on the electrochemical, photochromic, photovoltaic and redox properties of a variety of ruthenium complexes opens further perspectives of applications of ruthenium complexes, depending on their substitution pattern [303,304,305].

Sophisticated dendritic motifs have been made available for chemical sensors, electronic devices and catalytic reactions [306,307,308,309]. Multifunctional organometallic polymers obtained by ROMP in the presence of Mo and Ru complexes found interesting valorization in conducting materials and catalysis [310,311,312,313]. In a recent, very elegant work, it was demonstrated that mixed-valent “click” intertwined polymer units, containing biferrocenium chloride side chains, obtained by ROMP or radical chain reactions, form nanosnakes that encapsulate gold nanoparticles [314]. Worth notice is that polymers containing triazolylbiferrocenyl groups in the side or in the main chain, synthesized by ROMP, radical or “click” cycloaddition reactions, could be oxidized by [FeCp_2_][PF_6_], H[AuCl_4_], or Ag[BF_4_] to form stable triazolylbiferrocenium salts. Various Au and Ag nanoparticle networks were stabilized by these biferrocenium salts providing modified electrodes suitable for chemical sensing [315]. Elaborated bifunctional copolymers electrogenerated from pyrenebutyric acid, *N*α′,*N*α-bis(carboxymethyl)-l-lysine amide (NTA–pyrene) and, as a third component, the [tris-(2,2′-bipyridine)(4,4′-bis(4-pyrenyl-1-ylbutyloxy)-2,2′-bipyridine] ruthenium(II) hexafluorophosphate complex (Ru(II)–pyrene) allowed bioreceptor immobilization and transduction of biorecognition events. Such label-free photoelectrochemical immunosensors and aptasensors were applied to the determination of thrombin and the anti-cholera toxin antibody [316].

## 4. Ruthenium Complexes in Sustainable Processes

This brief account could not be concluded without signaling at least a part of the newly acquired relevance of ruthenium complexes for industrial valorization [317,318,319]. As seen from this overview, Ru complexes contribute to sustainability through conservation of fossil resources and energy. Thus, oleochemisty by means of ruthenium catalysis, a field of major interest and presently intensively explored, is producing value-added synthetic products such as olefins, polymer additives, surface coatings, pharmaceuticals, *etc.* from natural seed oil feedstocks largely available in many countries. By a metathesis-based technology, palm oil and jojoba oil can be used to make high performance olefins and other specialty chemicals for a wide range of applications, including surfactants, detergents, lubricants, and biofuels [320,321]. A representative example of metathesis starting from renewable raw materials is self-metathesis of methyl oleate and cross-metathesis of methyl oleate with (*Z*)-2-butene-1,4-diol diacetate promoted by a series of indenylidene Ru catalysts [322]. A versatile ring-closing metathesis process has been developed for the synthesis of polyamide (nylon) precursors of various chain-lengths (PA11, 12, and 13); the technology allows an economical and sustainable production of these polymers from oleic acid as a single starting material [323]. Furthermore, a highly chemoselective procedure for obtaining valuable alcohols and higher-oxidation-state compounds by catalytic *Z*-selective cross-metathesis holds promise for a future application on a large scale [324]. In a patented procedure for valorization of biomass materials, cellulose was saccharified using a Ru catalyst supported on carbonized cellulose containing SO_3_H groups to give glucose in a 40% yield, at 68% cellulose conversion [325]. Cross-metathesis of bio-sourced fatty nitriles with acrylonitrile provides an efficient way for conservation of natural resources [326].

Along quite different lines, alkenones, abundantly found in oceans, having chains of 37–39 carbon atoms, could be broken by olefin metathesis into 8–13 C-atom chains, thus becoming useful for jet fuel [327]. A newly disclosed invention employs Proxima (a thermoset resin for glass and carbon fiber reinforced composites, produced via olefin metathesis with Grubbs’ catalyst) to reduce the amount of carbon fiber required in hydrogen pressure vessels lowering in this way the cost of hydrogen storage tanks [328].

An important aspect in the industrial valorization of Ru complexes is the removal of ruthenium residues, mandatory in areas where the level of this heavy metal in the final products (pharmaceuticals, specialty polymers designed for food supplies, and biomedical, textile or electronic applications) could be problematic for human health [329]. Overall, the high potential and versatility of the Ru complexes attest to the importance they assume in chemistry and other scientific and technical areas and undoubtedly ensure the emergence, in the near future, of further developments serving mankind.

The valuable contributions from the authors of this Special Issue “**Ruthenium Complex**” of *Molecules* are highly appreciated. Special thanks are due to the reviewers of the papers as well as to the members of the editorial staff of *Molecules* for their initiative for this Special Issue and their kind cooperation during the editorial process. We hope the readers will benefit from the diverse facets of the chemistry and applications of ruthenium complexes, included in this Special Issue, that largely illustrate the newest trends in the field.
